# Novel Norrie disease gene mutations in Chinese patients with familial exudative vitreoretinopathy

**DOI:** 10.1186/s12886-021-01852-3

**Published:** 2021-02-15

**Authors:** Li-Yun Jia, Kai Ma

**Affiliations:** Beijing Tongren Hospital, Capital Medical University, Beijing, China

## Abstract

**Purpose:**

This study aims to analyze the Norrie disease gene (*NDP*) variants in patients with familial exudative vitreoretinopathy (FEVR) and their clinical features.

**Methods:**

Thirty-three Chinese patients (22 familial and 11 simplex) who were diagnosed as FEVR underwent detailed ocular examinations in Beijing Tongren Hospital. Peripheral venous blood was drawn from the patients and their family members for the extraction of genomic DNA. All exons of *NDP* gene were analyzed by direct sequencing of PCR-amplified DNA fragments.

**Results:**

Four novel mutations in *NDP* gene were identified in four X-linked FEVR families: a C → T transversion, c. 625C → T, in exon 3, resulting in a serine-to-proline change in codon 73 (S73P); a C → G transition, c. 751C → G, in exon 3, resulting in an arginine-to-glycine change in codon 115 (R115G); a T → C transversion of nucleotide 331 at 5’UTR in exon 2 (c.331 T → C); and a C → T transversion of the nucleotide 5 in intron 1 (IVS1 + 5C → T). The mutations were not present in the control group (*n* = 100).

**Conclusions:**

Our results extend the spectrum of *NDP* gene mutations. The mutations in the non-coding region of *NDP* may play a crucial role in the pathogenesis of FEVR.

## Introduction

Familial exudative vitreoretinopathy (FEVR) is an inherited vitreous retinal disease characterized by incomplete vascularization of the peripheral retina with secondary complications including dragged retina, exudation, vitreous hemorrhage, and retinal detachment [1, 2]. The severity of the disease varies from no apparent visual symptoms or impairment to complete blindness [3].

The inheritance pattern of FEVR is autosomal dominant, recessive, and X-linked. To date, genes involved in the pathogenesis of FEVR include *FZD4*, *NDP*, *LRP5*, *TSPAN12*, *ZNF408*, *KIF11*, and *CTNNB1* [4–9]. The Norrie disease gene (*NDP*) is located on chromosome Xp11.2–11.3 and encodes for the 133-amino-acid protein Norrin. Norrin and FZD4 (Frizzled-4) function as a high-affinity ligand-receptor pair, and Norrin together with the auxiliary component tetraspanin-12 (TSPAN12) induces FZD4 and lipoprotein receptor-related protein-5 (LRP5) dependent activation of classical Wnt pathway via promoting the recruitment of β-catenin (encoded by *CTNNB1*) into the nucleus [10–12]. Defects in Wnt signaling cascade affect ocular growth and development and play an important role in the pathological processes underlying both X-linked FEVR and Norrie disease (ND, MIM#310620) [4], which may present as pseudoglioma, mental retardation, or hearing loss. *KIF11* encodes a mitotic kinesin known as Eg5, while *ZNF409* encodes a zinc-finger homeobox protein, but their function in FEVR remains unclear.

Over 160 mutations of the *NDP* gene have been reported so far, most of which are missense mutations (about 50%), while others are deletion mutations (about 6%)(HGMD Professional 2017.3, http://www.hgmd.cf.ac). Some nonsense, splicing site, insertion, deletion-insertion, and regulatory region mutations are also reported in the *NDP* gene. Most patients with *NDP* gene mutations show the Norrie disease phenotype; only 5% of patients show FEVR. Mutation screening of the *NDP* gene in our Chinese patients with FEVR could, therefore, determine further enrich the spectrum and frequency of mutations in NDP-causing FEVR.

## Methods

### Study participants

Thirty-three patients with FEVR and their family members were recruited for this study at the Beijing Tongren Hospital, Department of Ophthalmology. Of the 33 patients, 22 had familial FEVR and 11 had sporadic FEVR (with no apparent family history). All the patients were Chinese and were born at term with normal weight. The diagnosis of FEVR was based on the presence of at least one of the typical clinical signs including peripheral retinal avascularization with abnormal retinal vascular formation, severe retinal exudates, retinal neovascularization, peripheral fibrovascular mass, macular ectopia, retinal folds, retinal detachment, and vitreous hemorrhage. Fundus fluorescein angiography was performed in the selected cases to confirm the diagnosis [13]. Genetically unrelated individuals from the Beijing Tongren Hospital were recruited as controls for conditions such as senile cataract, floaters, and itchy eyes. They underwent the same ophthalmic examinations and were diagnosed not to have FEVR or other major eye diseases [13]. The study protocol was approved by the Ethics Committee for Human Research of the Beijing Tongren Hospital Capital Medical University and adhered to the tenets of the Declaration of Helsinki. Informed consent was obtained from all the volunteers after providing a detailed explanation of the nature and possible consequences of the study. Venous blood was obtained from the participants and stored at − 20 °C for less than 2 months before DNA extraction.

### *NDP* screening

Genomic DNA was extracted from 200 μl of whole blood using a Qiamp Blood Kit (Qiagen, Hilden, Germany). To detect any possible novel disease-causing mutations in the *NDP* gene, the three coding exons and adjacent sequences of *NDP* were screened by polymerase chain reaction followed by direct DNA sequencing with the BigDye Terminator DNA sequencing kit on a 3130XL analyzer (Applied Biosystems, Foster City, CA, USA), using the same set of primers previously described by Chen et al. [4] Reference sequence of *NDP* (NM_012193.2; GenBank, http://www.ncbi.nlm.nih.gov/Genbank; provided in the public domain by the National Center for Biotechnology Information) was used for the identification of the variants.

## Results

**Fig. 1:**
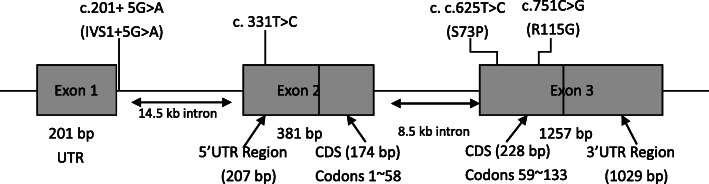
Schematic diagram of the *NDP* gene and locations of the mutations

**Fig. 2:**
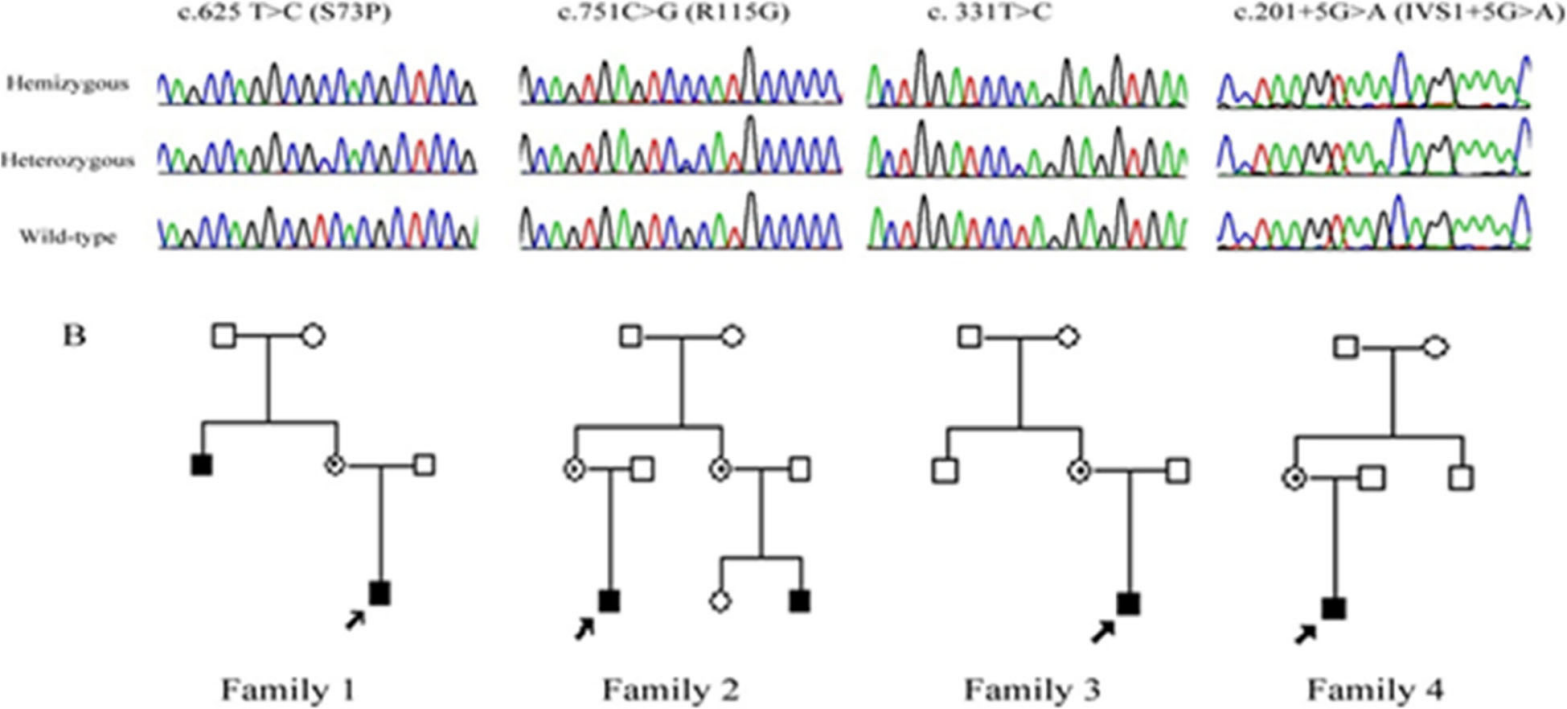
Schematic pedigrees of the families with *NDP* mutations. Arrows indicate probands; open symbols, clinically unaffected; solid symbols, clinically affected; diagonal lines through symbols, deceased; vertical lines in symbols, affected by hearsay; circles inside symbols, asymptomatic for familial exudative vitreoretinopathy; +/−, heterozygous with familial mutation; and −/−, wild type. For the *NDP* mutations, the nomenclature is based on GenBank NM_012193.2

The DNAs from the 33 FEVR patients were analyzed for mutations in the three exons and flanking exon-intron boundaries of *NDP* gene. Four different novel mutations were identified in four FEVR families (Table 1, and Figs. 1 and 2). All probands were men, and therefore, these changes were interpreted as homozygous. These changes in the sequence were not found in the 100 unrelated and unaffected individuals, all females, in the Chinese population. All the changes were co-segregated as the X-linked recessive form of FEVR in the four families in this study.

**Table 1:** Mutations in *NDP* gene and the associated clinical findings

Family^a^	DNA Chang (Protein Prediction)	ID^a^/Sex/Age	Allele Status	Visual Acuity (Refraction)	Vitreoretinal Findings	Comments
**1**	c.625 T → C in exon 3 (S73P)	Ш-1/M/11 mo	Homozygous	BE: follow a moving object	BE: Avascular retina, fibrous proliferation, dragged macula	
		П-1/M/30 y	Homozygous	RE: 0.3 (−5.0 D) LE: 0.4 (−4.5 D)	BE: Avascular retina, fibrous proliferation, persistent hyaloids remnant	PHC, encircling (BE)
		П-2/F/31 y	Heterozygous	RE: 1.2 (nc) LE: 1.2 (nc)	BE: Normal	
**2**	c.751C → G in exon 3 (R115G)	Ш-1/M/4 mo	Homozygous	RE: HM (nc) LE: follow a moving object	RE: RLF, RD LE: Avascular retina, fibrous proliferation, dragged macula, persistent hyaloids remnant	Lx (RE) PHC (LE)
		П-2/F/26 y	Heterozygous	RE: 1.5 (nc) LE: 1.5 (nc)	BE: Normal	
**3**	c.331 T → C in exon 2	Ш-2/M/6 mo	Homozygous	BE: follow a moving object	BE: RLF, dragged macula, persistent hyaloid remnant	PHC, encircling (BE)
		Ш-1/M/7 y	Homozygous	RE: NLP (nc) LE: LP (nc)	BE: RLF, RD	Vx Lx (BE)
		П-1/M/30 y	Homozygous	RE: NLP (nc) LE: NLP (nc)	BE: RLF, RD, flat anterior chamber	Lx (BE)
		П-3/F/24 y	Heterozygous	RE: 1.2 (nc) LE: 1.0 (nc)	BE: Normal	
		П-4/F/28 y	Heterozygous	RE: 1.2 (nc) LE: 1.2 (nc)	BE: Normal	
**4**	c.201 + 5G → A (IVS1 + 5G → A)	Ш-1/M/2 y	Homozygous	BE: follow a moving object	BE: Avascular retina, fibrous proliferation, dragged macula, persistent hyaloids remnant	PHC (BE)
		П-1/F/27 y	Heterozygous	RE: 1.5 (−4.0 D) LE: 1.5 (−4.0 D)	BE: Normal	

*RE* Right eye, *LE* Left eye, *BE* Both eyes, *LP* Light perception, *HM* Hand moving, *RLF* Retrolental fibroplasia, *RD* Retinal detachment, *PHC* Photocoagulation, *Vx* Vitrectomy, *Lx* Lensectomy

^a^ Identifications refer to Fig. 2

**Fig. 3:**
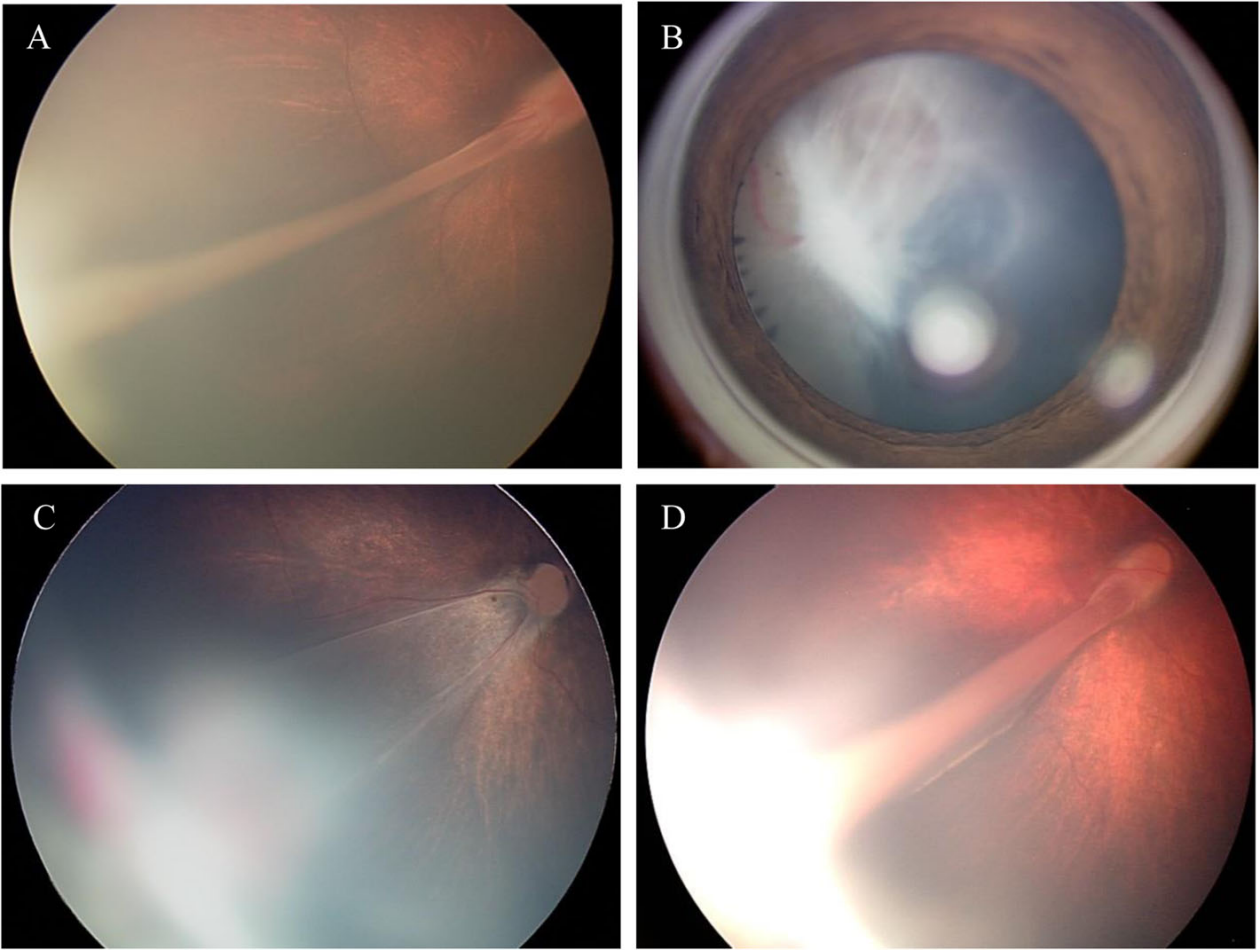
Fundus images of proband in FEVR families

Four members of Family 1 were studied. The proband was an 11-month-old boy who showed bilateral retinal folds resembling persistent hyperplastic primary vitreous (Fig. 3a). His maternal uncle was diagnosed with FEVR because of bilateral retinal folds at 7 years of age. Direct DNA sequencing of these two cases revealed a C → T transversion, c. 625C → T, in exon 3 of the *NDP* gene, where serine was replaced by proline at codon 73 (S73P; Fig. 2). The proband’s mother was heterozygous for the S73P mutation.

In Family 2, the proband was a 4-month-old boy who showed a retrolental mass with total retinal detachment in the right eye (Fig. 3b). The typical features of FEVR, such as peripheral avascularization temporal to the macula and neovascularization of the retinal vessels, were observed in the left eye where laser photocoagulation had been performed. Sequence analyses demonstrated that he carried a C → G transition, c. 751C → G, in exon 3, resulting in an arginine-to-glycine change in codon 115 (R115G; Fig. 2). None of the family members had FEVR, however, the mother was heterozygous for the R115G mutation.

Family 3 was a three-generation family with affected individuals presenting X-linked exudative vitreoretinopathy without deafness or mental retardation. A T → C transversion of nucleotide 331 at 5’UTR in exon 2 (c.331 T → C) of *NDP* gene was identified in all the affected family members. The proband was a 6-month-old boy who showed bilateral retinal folds, macular ectopia, retrolental fibroplasia, and retinal detachment (Fig. 3c). The proband’s maternal cousin (son of his mother’s sister) was diagnosed with FEVR resulting from retrolental fibroplasias and retinal detachment at the age of 7 years. His vision was restricted to light perception in the left eye but no light perception in the right eye, and he could not follow a moving finger with either eye. Furthermore, the proband’s maternal uncle, who was blind since the age of five, presented with bilateral retrolental fibroplasias, retinal detachment, and a flat anterior chamber.

In Family 4, the proband was a 2-year-old boy. He was referred to our hospital at 7 months of age, and FEVR was suspected because of bilateral retinal folds, macular ectopia, retrolental fibroplasias, and persistent hyaloid remnant (Fig. 3d). Photocoagulation was performed on both eyes at the age of 1 year, after which he could follow a moving object. Sequence analyses demonstrated that he carried a C → T transversion of nucleotide five in intron 1 (IVS1 + 5C → T) (Fig. 2). Although both parents did not have any ocular abnormalities, the mother was heterozygous for the IVS1 + 5C → T mutation.

## Discussion

We identified four novel disease-causing mutations in the *NDP* gene in four different Chinese families. Of the four mutations, one was a novel splicing mutation, one was a novel 5’UTR mutation, while the other two were novel missense mutations. All of these mutations were responsible for X-linked FEVR and were not found in normal individuals from the Chinese population. These results suggest that the four mutations are pathogenic. Furthermore, our data indicate that the *NDP* mutations cause about 12.1% (4/33) of FEVR in the Chinese population. The prevalence of *NDP* mutation in our patients is slightly higher than that reported in previous studies, which may be due to ethnic difference.

*NDP* gene-related diseases are X-linked recessive genetic diseases [4, 14]. *NDP*-related retinal diseases, caused by abnormalities in the *NDP* gene, include various progressive diseases with retinal vascular hypoplasia, such as Norrie disease, Coat’s disease, FEVR, persistent hyperplastic primary vitreous, and retinopathy of prematurity [14–17].

The *NDP* gene spans 2.8 kBP, and the cDNA contains three exons. Exon 1 does not encode and may promote gene transcription [18]. The region encoding the protein is located in the second half of the second exon and the first part of the third exon. Previous studies have shown that most of the *NDP* gene mutations leading to the FEVR phenotype are missense mutations, while the nonsense, splice-site, and large deletion mutations mainly lead to Norrie disease. To date, nearly 20 mutations in the *NDP* gene have been identified in individuals with X-linked familial and sporadic exudative vitreoretinopathy, however, all of them were missense mutations. The missense mutations may lead to drastic amino acid changes and often affect one of the many cysteine residues immediately adjacent to a cysteine. Mutations in these regions may disrupt protein folding or directly interfere with the norrin-receptor interactions [5, 14, 18, 19]. Schubak et al. detected *NDP* gene mutations in 24 families, most of which were located in or near cysteine residues [20]. Furthermore, Wu et al. [19]. analyzed 11 patients with *NDP* gene mutations, of whom, four with Norrie disease had *NDP* mutations involving cysteine residues, while four with FEVR, one with retinopathy of prematurity, and one with persistent primary vitreous hyperplasia did not [21]. However, in our study, two novel missense mutations (S73P and R115G) did not involve cysteine.

Two patients with the novel missense mutations, S73P and R115G, had typical manifestations of FEVR, including peripheral retinal vascular anomalies, fibrous proliferation, and dragged macula. This finding is consistent with that in an mice model with an *NDP* mutation [22]. Multiple sequence alignment of human Norrin against the available species sequences revealed that codons S73 and R115 were conserved in rats and mice [data not shown]. S73P and R115G were the non-conserved mutations located within the cystine knot domain. Moreover, Walker et al. [23] previously reported one family with Norrie disease with a 626C → A transversion of the *NDP* gene, resulting in an S73X substitution. The affected family members had a severe ocular phenotype, but no mental retardation or deafness. In addition, the R115L mutation was previously identified by Kondo and coworkers in a Japanese patient with typical FEVR [24]. This finding is consistent with that of Wu et al. [19] who reported the mutations in the non-cysteine residues of the Norrin protein that led to abnormal vascular and retinal development and phenotypes consistent with FEVR. Using a Norrin-based reporter assay to analyze the effects of the FEVR-causing mutations, Qin et al. [25] demonstrated that Norrin mutants (K54N and R115L) demonstrated variable effects on signal transduction and impaired cell surface binding. Although the identification of a second point mutation affecting amino acid 73 and 115 in these FEVR families made the proposed pathogenic nature of the S73P and R115G robust, the functional relevance of each mutation should be explored in further detail.

In this study, in Family 3, a T → C transversion of nucleotide 331 at 5’UTR in exon 2 (c.331 T → C) of the *NDP* gene was identified in all the three affected members, whereas, in Family 4, a C → T transversion of nucleotide five in intron 1 (IVS1 + 5C → T) was identified in a 2-years-old boy with FEVR. In addition, no pathogenic mutation was found in the subsequent screening of other genes related to FEVR (*FZD4*, *LRP5*, *TSPAN12*, *ZNF408*, and *CTNNB1*) [data not shown]. Although the two mutations (c.331 T → C and IVS1 + 5C → T) belonged to the non-coding region of the *NDP* gene, we believe that they are pathogenic. Ghislaine et al. [14] previously reported two French families with Norrie disease with a c.1A → G in exon 1 in an 18-month-old boy and a c.23479–1G → C in intron 2 in the families of five patients. These data indicate that the mutations in the non-coding region may still lead to protein misfolding, resulting in functional abnormalities that may lead to various diseases.

In summary, we identified four novel mutations that will extend spectrum on *NDP* mutations, especially in Chinese FEVR patients. The sequence changes of *NDP* gene that do not cause changes in the amino acid sequence may play a role in the pathogenesis of FEVR. Patients with *NDP* mutations have the characteristics of younger onset age and serious eye disease.

## Data Availability

All data generated or analysed during this study are included in this published article.
